# Titanate–polyurethane–chitosan ternary nanocomposite as an efficient coating for steel against corrosion

**DOI:** 10.1038/s41598-024-81104-8

**Published:** 2024-12-19

**Authors:** Ahmed S. Abdellatif, Mohammed Shahien, Ashraf M. El-Saeed, Ayman H. Zaki

**Affiliations:** 1 Materials Science and Nanotechnology Department, Faculty of Postgraduate Studies for Advanced Sciences (PSAS), Beni-Suef University, Beni-Suef, 62511 Egypt; 2 Advanced Manufacturing Research Institute (AMRI), National Institute of Advanced Industrial Science and Technology (AIST), 305-8564 Tsukuba, Japan; 3 Advanced Materials Institute, Central Metallurgical R&D Institute (CMRDI), Helwan, Cairo Egypt; 4 Petroleum applications department, Egyptian Petroleum Research Institute (EPRI), Nasr City11727, Cairo, Egypt

**Keywords:** Nanotubes, Corrosion, Drop coating, Titanate, Polymer, Materials science, Nanoscience and technology

## Abstract

In this study, a titanate–polyurethane–chitosan ternary nanocomposite was prepared by physical mixing. Sodium titanate nanotubes (Na-TNTs) were prepared by the hydrothermal method, and chitosan was extracted from shrimp shell. Na-TNTs were mixed with polyurethane (PU) of different ratios by weight, and chitosan was added after optimization. All of the nanocomposite samples were characterized by field-emission scanning electron microscopy (FESEM), and the mechanical properties were investigated by abrasion, adhesion pull-off, impact resistance, and T-bending tests. The anticorrosion ability was tested by the salt spray method. The obtained results revealed that the binary composite of PU and 1.5% Na-TNTs exhibited remarkable anticorrosion activity among all the binary composites where the disbonded area 5% compared to blank PU 19% and adhesion 5.1 MPa compared to blank 3.5 MPa, while the ternary composite containing 4% chitosan exhibited the best anticorrosion activity where the disbonded area 2% and also exhibit better adhesion 7.9 MPa.

## Introduction

Metals and alloys are important for several technological and industrial applications. Nevertheless, their applications under humid conditions lead to corrosion. Corrosion causes chemical changes in the metallic surfaces, leading to changes in the appearance and structure of metals; these changes may cause major injuries or death in case of metallic bridges. The global corrosion cost was estimated to be 3 billion in 2022. Corrosion is unavoidable; however, it can be reduced by surface coating, which increases the service life and reduces maintenance costs. The prevailing consensus is that coatings create a barrier that effectively hinders the flow of oxygen and water, thereby intensifying the hindrance of the movement of ions at the interface between the metal and electrolyte^1^. Typically, urethanes, latex, epoxy, and silicone alkyd are used as coatings to prevent corrosion^2^. Owing to their high mechanical strength, resistance, good stability against thermal, and chemical resistance, polyurethane (PU) is utilized in a wide range of applications and industries, such as the building, construction, transportation, and automotive industries^3,4^.

Nano material used as fillers in organic coating, The addition of nanoparticles to the polymeric matrix considerably affects the properties of polymers, can significantly enhance the performance and properties of this coating, The choice of nanoparticles to be applied for coating modification depends on the specific property to be tailored. For example, Titanium oxide nanotubes^15^, single-walled carbon nanotubes and multi-walled carbon nanotubes^2^.

Titanium oxide nanotubes (TiO_2_ nanotubes, TNT) represent a highly promising class of nanostructured oxides characterized by their tubular morphology. These materials are cost-effective, exhibit chemical stability, and are non-toxic^15^.

SWCNTs and MWCNTs) have emerged as highly favored nanomaterials for corrosion-resistant coatings, which have been employed extensively either as reinforcement or for imparting specific functional properties^2^. A nanocomposite comprising a conductive PU and 5.0 wt% MWNTs was reported to improve thermal stability^1^.

Titanate nanotubes (TNTs) have garnered increasing interest and are emerging as a compelling option across various disciplines. Their remarkable performance and promising outcomes contribute significant advantages to fields that incorporate them, including green chemistry and engineering. Titanate nanotubes (TNTs) represent a category of innovative materials that were first synthesized in 1996, following the introduction of organic carbon nanotubes in 1991, which demonstrated promising applications across various fields. TNTs are characterized as spiral-shaped inorganic nanoparticles, originating from titanium dioxide (TiO_2_) as the primary raw material. The diameter of these nanotubes generally falls within the range of 10 to 100 nm, depending on the specific preparation technique employed; however, in certain instances, it can extend up to 400 nm. This nanoscale dimension, coupled with their tubular architecture, constitutes distinctive features that set them apart from other forms of nanoparticles (NPs)^40^. TNTs possess an increased surface area and enhanced cellular uptake capabilities. Furthermore, they exhibit a unique set of characteristics that may be advantageous across various domains. These include their hydrophilic properties, favorable wettability, biocompatibility, chemical stability, superior mechanical strength, and resistance to corrosion, coupled with low toxicity levels^40^.

For several years, chromates have been used as a protective coating for steels, functioning via the inhibition mechanism, but their toxicity hinders their applications^5^ Hence, replacing this inorganic toxic inhibitor by an organic nontoxic corrosion inhibitor such as chitosan, which is a new type of a self-healing protective coating, is imperative^6,7^. Chitosan represents the most abundant and renewable marine polymer, distinguished as a highly promising eco-friendly option for surface coating applications. Its remarkable film-forming capabilities, coupled with its corrosion resistance attributed to its barrier properties and strong affinity for metals, enhance its suitability for such uses^6^. Chitosan exhibits excellent film-forming abilities and superior adherence to numerous organic polymers and metallic surfaces, as well as the capacity to form reversible complexes with possible corrosion inhibitors^8^.

This study aims to develop a novel titanate-polyurethane-chitosan nanocomposite coating to enhance the corrosion resistance and mechanical properties of steel, while maintaining environmental safety and cost-efficiency. sodium titanate was added to PU for the first time to enhance the corrosion resistance, and mechanical properties of PU. In addition, chitosan as a natural biopolymer was added to the prepared composites to considerably improve the aforementioned properties. Using of titanate nanotube and chitosan together as a filler of PU is a novel to collect between properties both of them like barrier properties, flexibility, adhesion, self-healing, corrosion resistance and low cost.

## Materials and methods

### Materials

Anatase TiO_2_ powder (Loba Chemie Pvt. Ltd., India) and NaOH (Al-Nasr Company, Egypt) were used as the starting raw materials. Acrylic PU SIGMADUR TM 550 (Sigma Paints, PPG Industries, Egypt), comprising a hydroxyl-functionalized acrylic-styrene reason with a viscosity of 25–35 mPa⋅s, a solid content of 61–95%, and an aliphatic polyisocyanate as the hardener with a viscosity of 250 ± 75 mPa⋅s and a non-volatile content of 75 ± 1 was used.

### Methods and techniques

#### Synthesis of sodium titanate nanotubes (Na_2_Ti_3_O_7_)

Sodium titanate nanotubes were prepared by the hydrothermal method, the details of which were reported elsewhere^9–13^. First, as-received titanium dioxide powder (10 g) was dissolved in 500 mL of a 10 M NaOH solution with continuous stirring for approximately 30 min, affording a milky suspension. Second, the suspension was added into a Teflon-lined autoclave and dried at 160 °C for 23 h. After cooling the powder, it was washed several times using distilled water to remove residual NaOH. Finally, the synthesized powder was dried at 80 °C for 12 h, which was referred to as sodium titanate nanotubes (Na-TNTs).

#### Chitosan extraction

Chitin was extracted from locally purchased shrimp shells, and chitosan was obtained by the alkali deacetylation of chitin as follows: First, shrimp shells were deproteinized using a 3.5% (w/w) NaOH solution at 65 °C for 2 h in a solid-to-liquid ratio of 1:10 (w/v); this process was repeated three times to obtain white shrimp. Then, shrimp was demineralized using HCl (1 N) at an ambient temperature for 1 day, decolorized using acetone at 50 °C for 2 h, and dried at room temperature for 2 h. To remove the acetyl groups from the recovered chitin, chitin was combined with a 50% NaOH solution and agitated at 115 °C for 2 h in a solid-to-solvent ratio of 1:10 (w/v). The obtained chitosan was washed under flowing tap water until it was neutral, rinsed with distilled water, filtered, and dried at 60 °C for 24 h, the details of which were reported elsewhere^6^.

#### Fabrication of a polyurethane-sodium titanate nanotube coating

The aliphatic acrylic PU resin was prepared by mixing the hydroxyl-functionalized acrylic-styrene resin with an aliphatic polyisocyanate as the hardener in a mixing ratio of 88:12 to coat the carbon steel alloy plate as a blank sample at room temperature without induction time. Sodium titanate nanotubes were mixed with the acrylic PU resin in different ratios of 0.5, 1, 1.5, 2, and 2.5 wt% (based on the total weight of acrylic PU and the hardener). The prepared Na-TNT powder was mixed with the PU resin and sonicated for 25 min, and the mixture was magnetically stirred for 1 h. Then, the hardener was added gradually to the mixture under continuous stirring at room temperature for 30 min to initiate curing. The prepared mixture of the acrylic PU–sodium titanate nanotubes was applied to the surface of the carbon steel plates in the same way as blank sample, and the films were dried at room temperature for 7 days to ensure that the films were completely cured and covered the surface.

#### Synthesis of polyurethane–chitosan–sodium titanate nanotube composite coating

First, 1.5% Na-TNTs were mixed with a thinner as the solvent, and the mixture was sonicated for 20 min. Next, chitosan with different ratio (1, 2, 3, 4 and 5%) was added to the formed suspension and stirred at a speed of up to 900 rpm for 30 min. Besides, the PU resin, and hardener were diluted separately using the thinner. Third, mixtures of Na-TNTS and chitosan were added to a resin–thinner solution under continuous stirring at a speed of up to 600 rpm for 15 min, and the hardener thinner solution was added to the mixture and stirred for 20 min. Finally, the coating mixture was applied to the surface-prepared plates, the surfaces were prepared for coating by power tool cleaning to pare metal, SSPC SP 11, according to the society for protective coating, all fabrication procedure implemented at room temperature. After 7 days, the completely cured samples were characterized to evaluate the effect of the chitosan ratio.

The experimental observations result from salt spray test revealed that using 1.5% Na-TNTs exhibited a significant effect as the disbonded area decreased from 19% in blank PU to 5%; based on these findings 1.5% Na-TNTs was selected as the optimum ratio, followed by the addition of different ratios of chitosan.

### Characterization

#### Characterization of sodium titanate nanotubes

Field-emission scanning electron microscopy (FESEM, Zeiss Sigma 500 VP, Germany) equipped with an energy-dispersive X-ray spectroscopy (EDX) detector was used for microstructure analysis, and some samples with (FESEM, Quanta FEG 250, Switzerland. The phase of was identified using a Panalytical X-ray diffractometer (Empyrean, Panalytical Ltd., UK). Transmission electron microscopy (TEM) was employed for microstructural analysis (JEOL, JEM-2100 F, Japan), and Fourier transform infrared (FTIR) spectra were recorded on a VERTEX 70 spectrophotometer.

### Evaluation of polyurethane nanocomposite-coated panels

#### Mechanical tests

Mechanical tests were conducted using ASTM procedures for organic coating on steel as follows: abrasion test (ASTM D4060-07), adhesion pull-off (ASTM D4541), impact resistance (ASTM D5420), and T-bending (ASTM D522).

#### Corrosion tests

The salt spray corrosion test was conducted according to ASTM B117 standards. Salt spray test conditions were 35 °C (± 1.5 °C), 95 ± 5% humidity, and 5% weight% of aqueous NaCl solution. The back side of test panels was protected against corrosive species and coated by using paraffin wax on the edges and on the uncoated back surface. An (X) was scribed to steel bare of each test panel. The length of each scribe line was approx. (10 cm) long. The salt-spray test results of the were assessed by two methods. The first one was due to visual inspection of blistering and rusting that appear on surface of panels. The other method was to evaluate the un-scribed areas of the test panels according to ASTM D1654 rating system. The test was performed in a chemically inert cabinet with a close-fitting lid. A salt mist was created by spraying through an atomizer. The panels were supported on non-metallic racks. The spray was so arranged that it did not impinge directly onto the panel surfaces. Panels were examined after 170 h and 500 h and. Before evaluation, coated panels were rinsed in running faucet water and dried.

## Results and discussion

### Dimensions and morphology of Na-TNTs

**Fig. 1:**
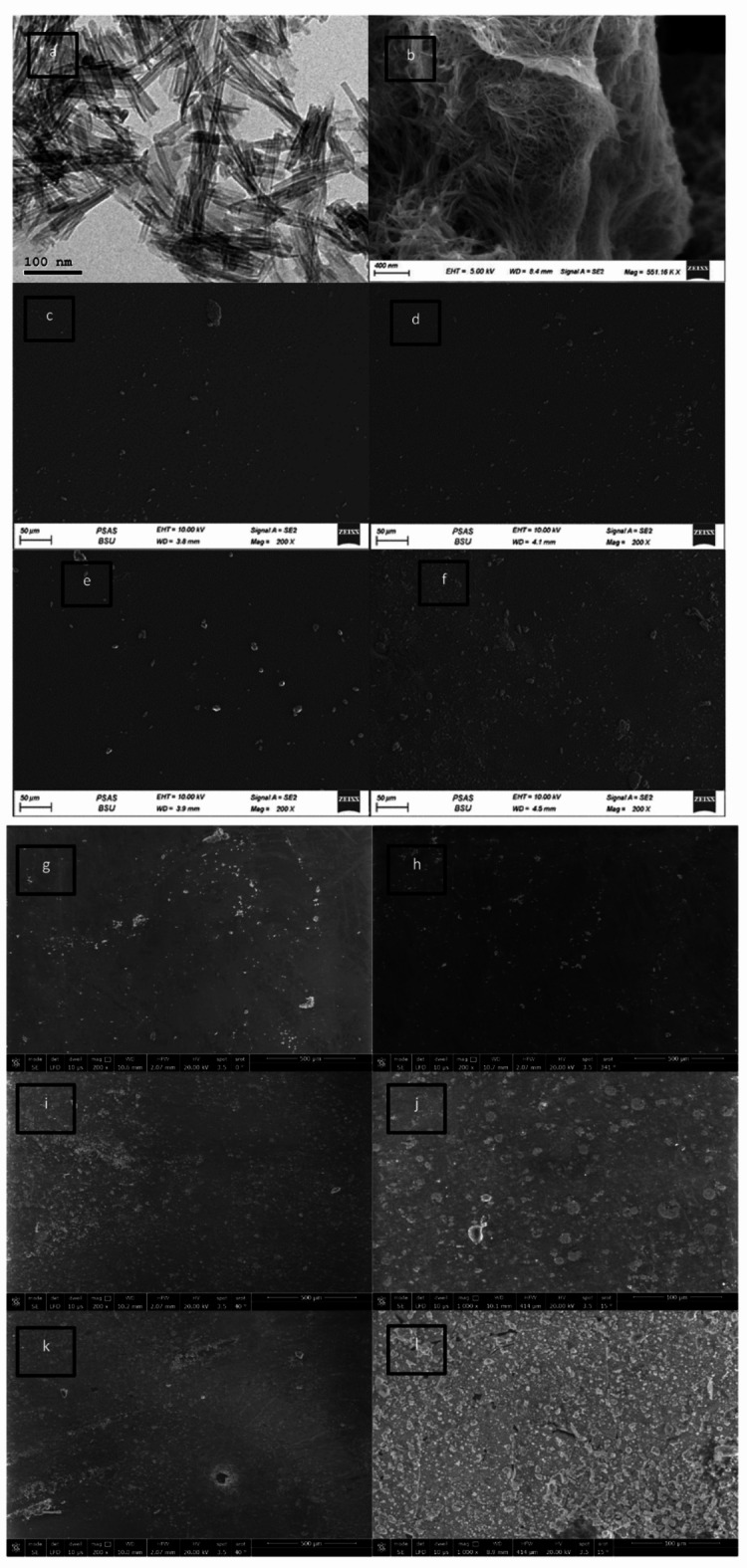
HRTEM and FESEM images of Na-TNTs (**a**, **b**, respectively). FESEM images of nanocomposites of polyurethane–1.5% Na-TNTs–chitosan with different contents (%), (**c**) 1% chitosan, (**d**) 2% chitosan, **e**) 3% chitosan, (**f**) 4% chitosan, and (**g**, **h**)0.5%. FESEM images of composites after salt spray, sample containing 4% chitosan (**i**, **j**), 5% chitosan (**k**, **l**).

Figure 1 (a), and (b) shows the HRTEM images, and FESEM images of the synthesized Na-TNTs powders, The microstructural images revealed the successful formation of the tubular nanosized Na-TNTs with an average diameter of about 11 nm Fig. 1 (a). while The FESEM monograph shows the formation of agglomerated, and randomly oriented-nanotubes Fig. 1 (b). This observation was in good agreement with that reported previously^14^.

### Characterization of the prepared coating films

Figure 1 (c, d, e, f, g, and h) shows the FESEM images of polyurethane coating containing 1.5% Na-TNTs and chitosan with different ratios 1, 2, 3, 4 and 5% respectively. This result indicated that by the addition of chitosan, the distribution of chitosan on the PU matrix increased significantly. While, Fig. 1 ( i, j, k, and l) shows the FESEM images of the composites containing 4% ( i, j), and 5% (k, l) chitosan, after corrosion, respectively. The images revealed that the sample containing 5% chitosan dramatically changed after salt spray test, where many holes and cracks can be observed, while the sample containing 4% chitosan showed no cracks or hole.

**Fig. 2:**
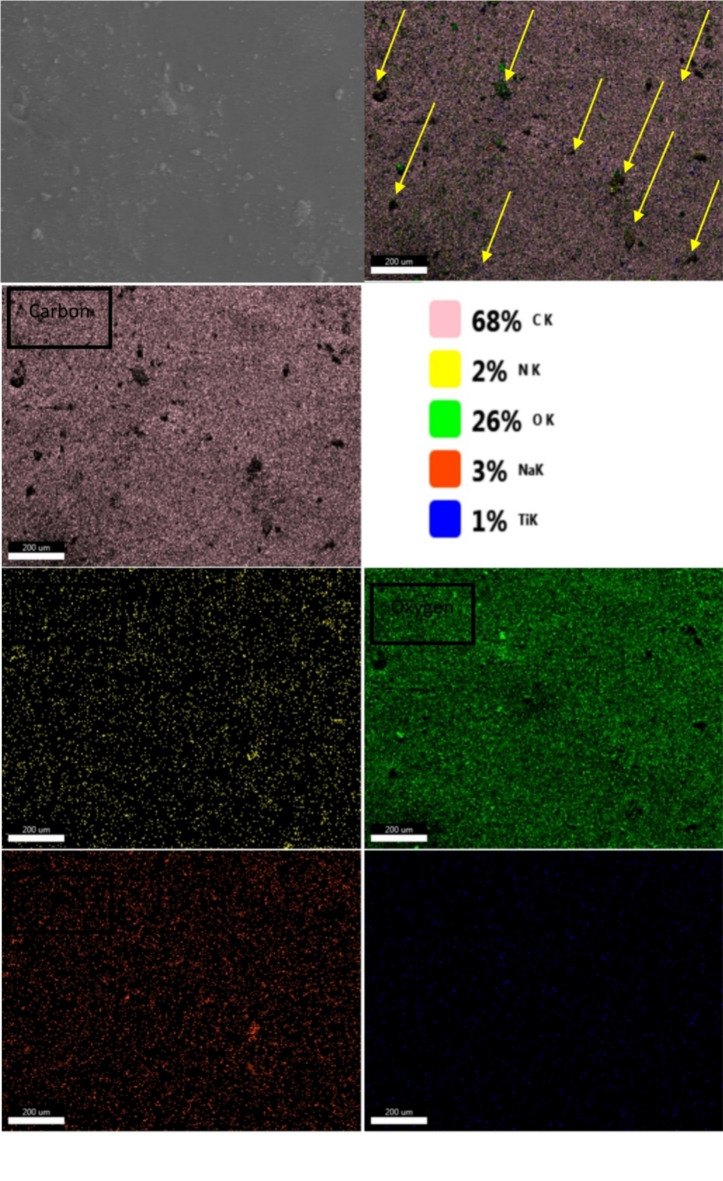
EDX mapping images of the composite containing 4% chitosan (polyurethane–1.5% Na-TNTs–4% chitosan).

Figure 2 shows the EDX mapping images of PU–Na-TNTs–4% chitosan: N, Ti, and Na were close to each other (as indicated by yellow arrows); this result was probably attributed to the formation of titanate–chitosan clusters and its uniform distribution in the PU matrix. Thereby, according to the FESEM images shown in Fig. 1 (c, d, e, f, g, and h), with the increase in the chitosan content, the number of the chitosan–titanate clusters increased in the PU matrix.

### Mechanical properties of the prepared nanocomposites

**Fig. 3:**
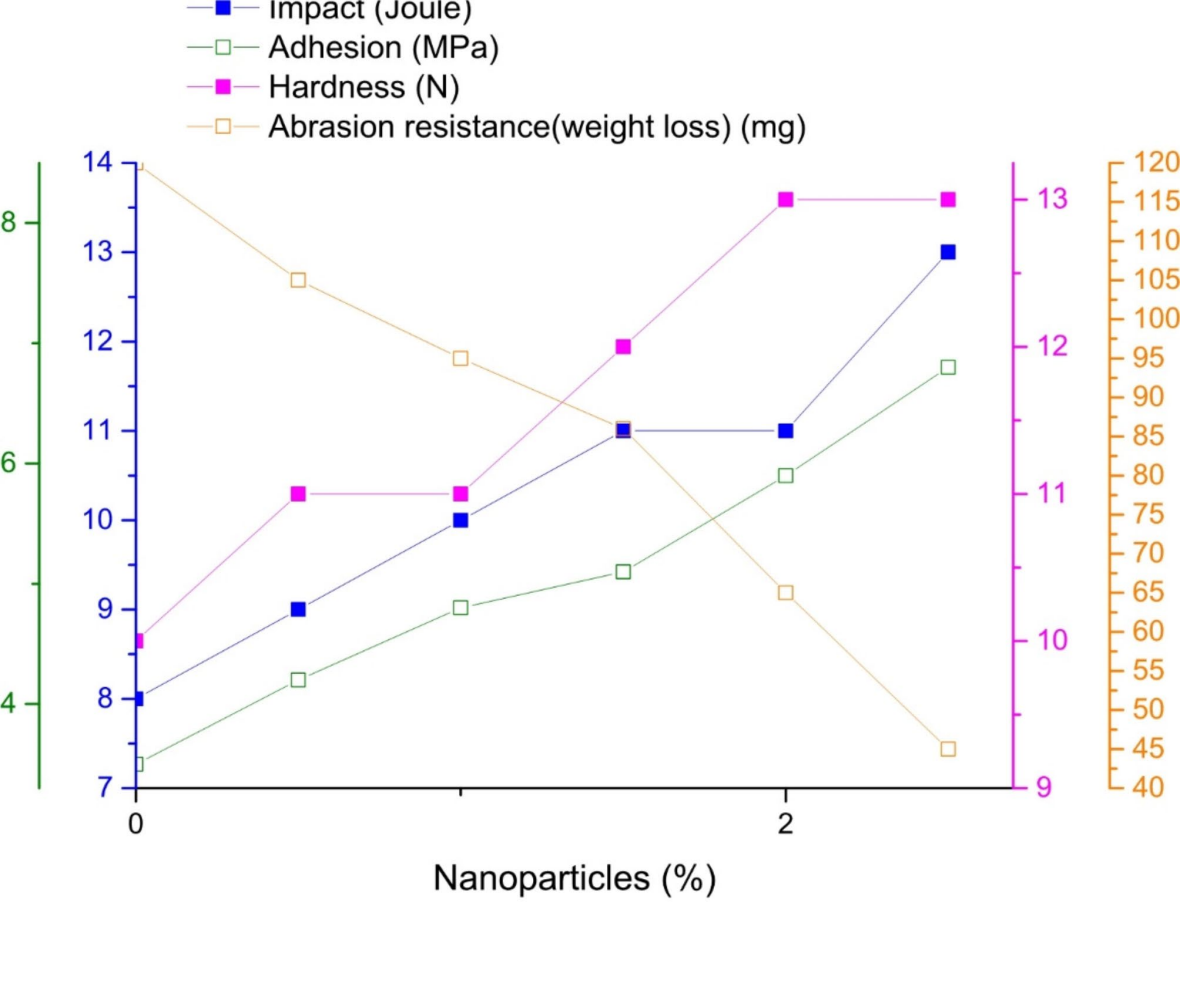
Mechanical properties of PU with different ratios of sodium-titanate nanotubes.

**Fig. 4:**
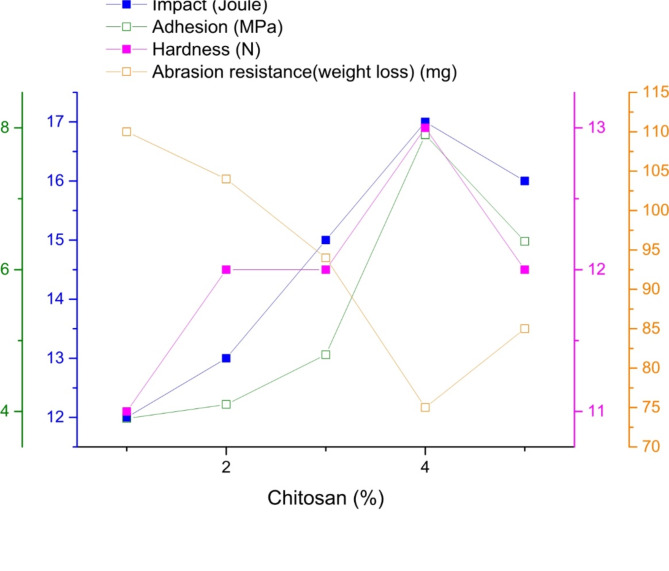
Mechanical properties of PU–Na-TNTs with different ratios of chitosan.

The mechanical properties of the cured blank PU and modified PU (addition of nanoparticles and chitosan), such as adhesion strength, impact resistance, hardness, bending, and abrasion, revealed that the addition of Na-TNTs and chitosan significantly improved the properties. Table 1; Fig. 3 show the mechanical properties of PU as function of the nanoparticle content, Table 2 ad Fig. 4 show the mechanical properties of PU–Na-TNTs as a function of the chitosan content. The observations of each test are separately described as follows:

i. After the addition of Na-TNTs in PU, the adhesion force between PU and the steel substrate increased from 3.5 MPa for blank PU to 6.5 MPa for PU with 2.5% Na-TNTs. The addition of chitosan led to the gradual increase in the adhesion strength, with the highest value of 7.9 MPa obtained with 4% chitosan, and then the adhesion strength started to decrease with 5% chitosan. The increase may be attributed to the presence of OH, which increased the adhesion between PU and chitosan^3^.
ii. By the addition of Na-TNTs in blank PU, the resistance to mechanical damage (impact test) for PU was improved from 8 J for blank PU to 13 J for PU with 2.5% Na-TNTs, and by the addition of 4% chitosan, it increased further to the maximum value of 17 J.
iii. By the addition of Na-TNTs, the scratch hardness was improved from 10.5 N for blank PU to 13 N for PU with 2% and 2.5% Na-TNTs. On the other hand, the addition of chitosan did not render further improvement, and a scratch hardness of 13 N was obtained with 4% chitosan.
iv. By the addition of Na-TNTs, the abrasion was improved from 120 mg for blank PU to 43 mg for PU with 2.5% Na-TNTs. Furthermore, the addition of chitosan into the binary composite was vital for improving abrasion resistance, and a better value was obtained with 4% chitosan.
v. All samples passed the bending test.

In this study, by the addition of well-distributed Na-TNTs in the PU matrix, the mechanical properties were enhanced. The efficacy of TNTs in improving mechanical characteristics is mostly attributed to their capacity for molecular interaction with the PU matrix^38,39^. The tubular shape of titanate will reinforce the film by forming cross linked network, This reinforcement improved the mechanical properties as showed in result of Table 1; Fig. 3. By the addition of chitosan, the chain order of the PU network increased due to the presence of chitosan particles. Hence, changes in the mechanical properties of the composite in comparison to pure PU and the extent and direction of these changes were significantly related to the chitosan amount and the degree of their interaction with the PU chains^5,7^. Recently, Piotrowska-Kirschling reported that the mechanical properties of PU–chitosan composites were lower than those of pure PU. However, they reported that in some instances, the use of low amounts of chitosan caused some improvement^3^, indicative of non-continuous improvement in the mechanical properties at high concentrations. The presence of agglomerated chitosan result in decreasing mechanical properties of the composite. This is because large clusters can act as stress concentrators, leading to premature failure^35,36^. The optimum result of mechanical properties due good distribution on the surface. When chitosan is well-dispersed, it can penetrate into surface irregularities of the substrate, creating mechanical interlocking. This physical entanglement between the chitosan and the substrate further strengthens adhesion 7.9 MPa, as it increases the effective contact area between the two materials^3^. As the amount of chitosan in a coating rises, especially when surpassing specific limits (such as 5 wt%), agglomeration takes place, resulting in phase separation. This phenomenon leads to an uneven distribution of chitosan throughout the matrix, which may negatively impact the mechanical characteristics of the coating^3,41^.

**Table 1:** Mechanical evaluation of blank polyurethane and Sodium Titanate nanotubes coated panels.

Nanoparticles %	Impact (Joule)	Adhesion (MPa)	Bending	Hardness (*N*)	Abrasion resistance (weight loss) mg / 1000cycle
Blank polyurethane	8	3.5	pass	10	**120**
0.5	9	4.2	pass	11	**105**
1.0	10	4.8	pass	11	**95**
1.5	11	5.1	pass	12	**86**
2.0	11	5.9	pass	13	**65**
2.5	13	6.8	pass	13	**45**

**Table 2:** Mechanical evaluation of polyurethane and chitosan coated panels, containing 1.5% Na-TNTs.

Chitosan %	Impact (Joule)	Adhesion (MPa)	Bending	Hardness	Abrasion resistance (weight loss)
1	12	3.9	pass	11	**110**
2	13	4.1	pass	12	**104**
3	15	4.8	pass	12	**94**
4	17	7.9	pass	13	**75**
5	16	6.4	pass	12	**85**

### Corrosion resistance of the prepared nanocomposites

**Fig. 5:**
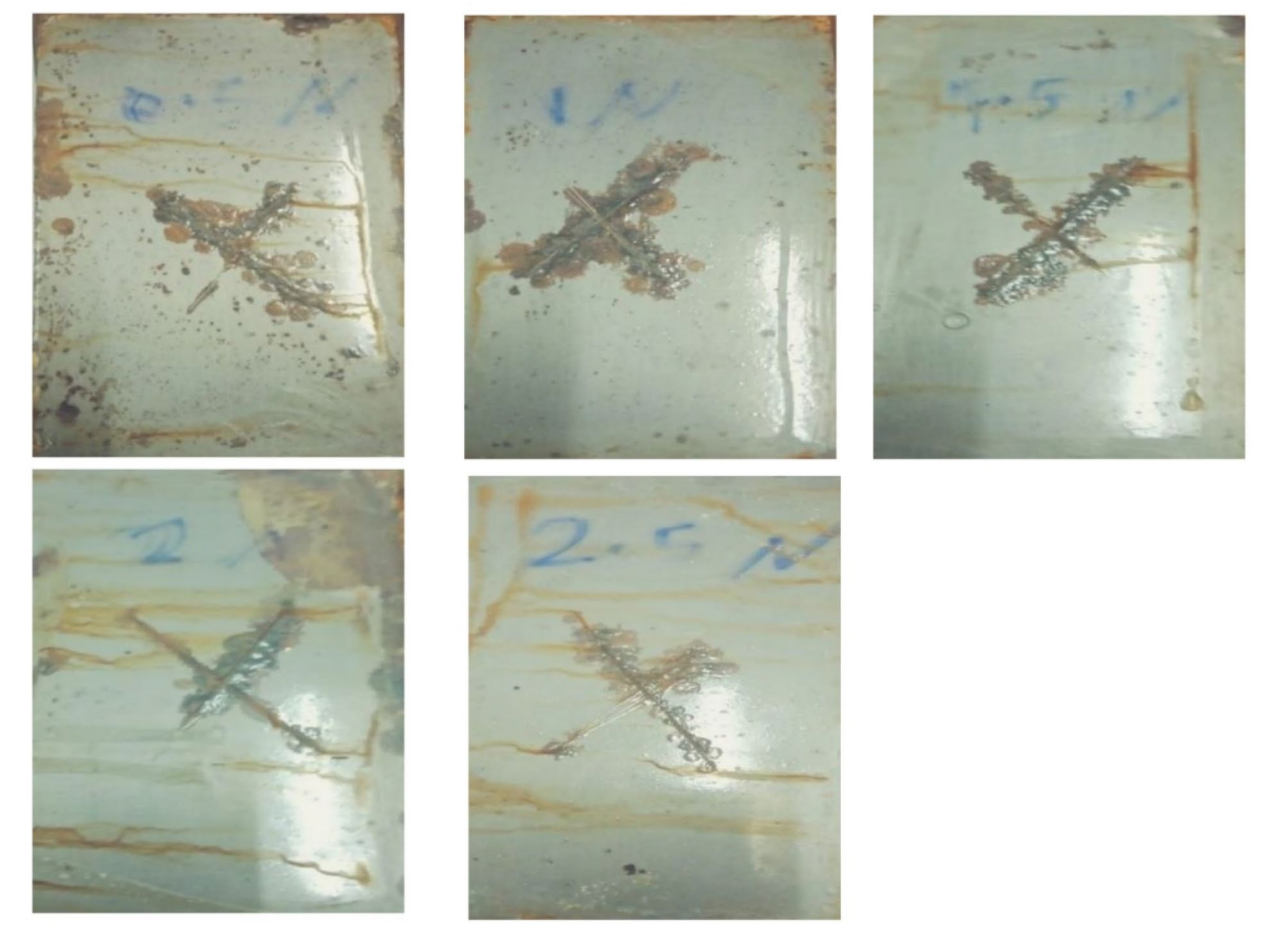
Salt spray tests of Pu–Na-TNT composites.

**Fig. 6:**
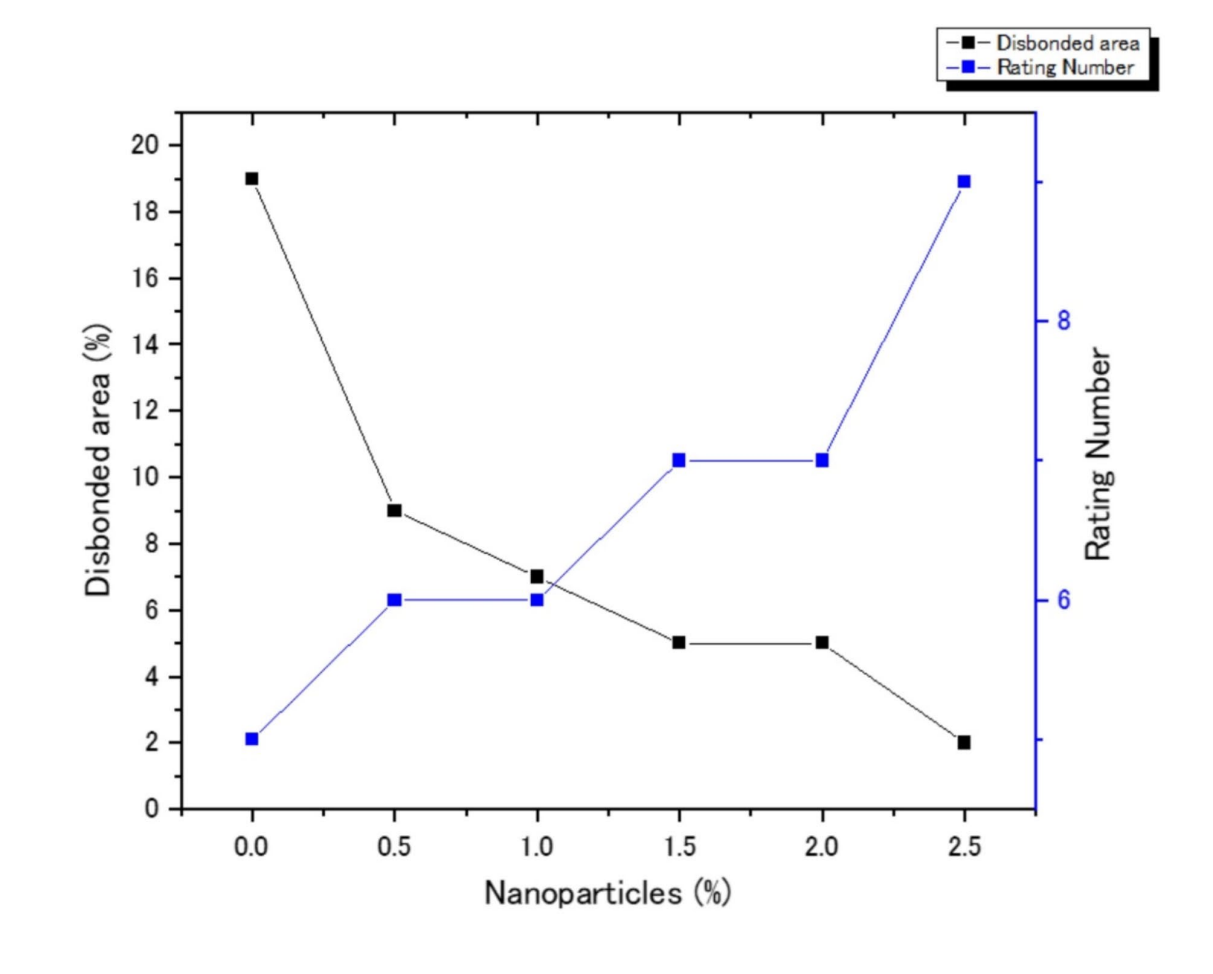
Effect of the content of Na-TNTs (%) on the disbonded area.

**Fig. 7:**
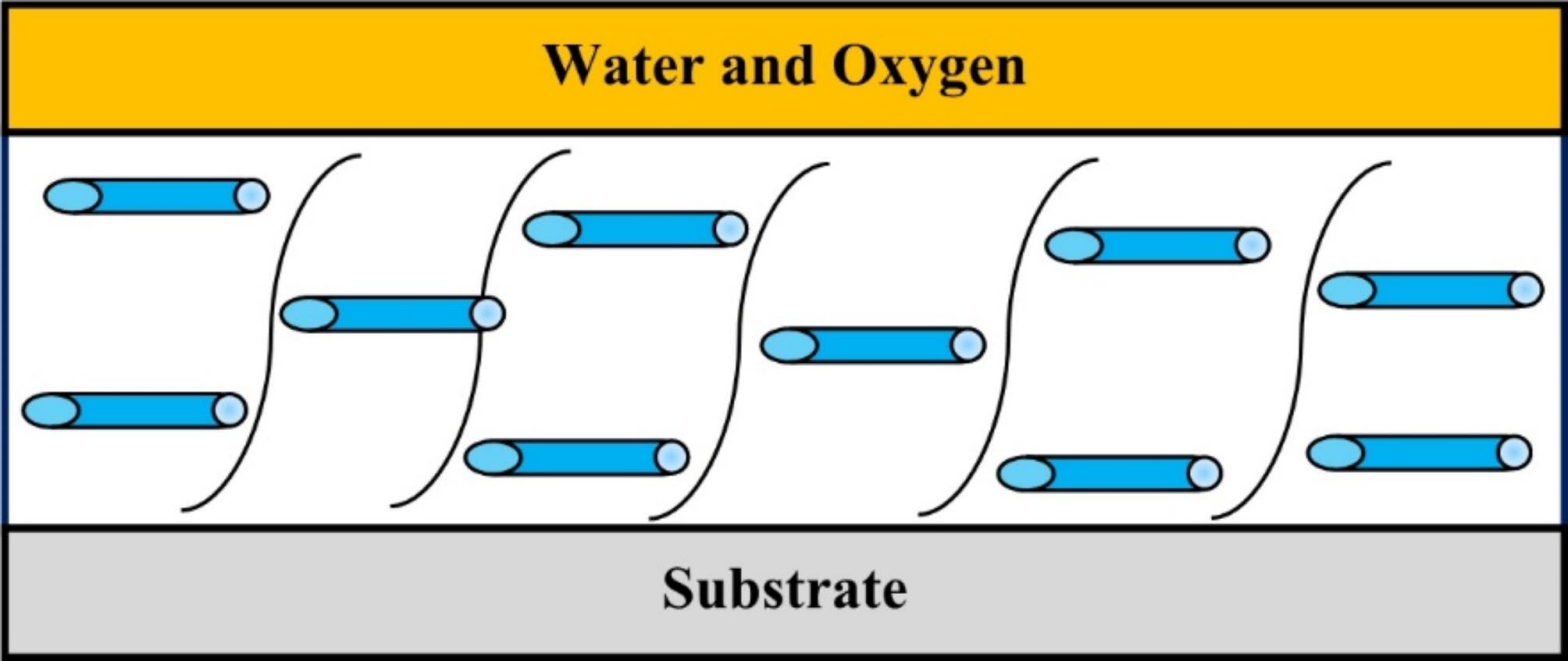
Schematic of the corrosion inhibition mechanism using the sodium-titanate nanotubes.

Figure 5 shows the corrosion behaviors of PU–Na-TNT composites by Salt spray tests, The evaluation was implemented according to ASTM D 1654, the obtained results are recorded in Table 3 and represented in Fig. 6. The results indicated that by the addition of titanate nanotubes, percentage of the disbonded area decreased from 19% for PU to 5% by the addition of 1.5% and 2% Na-TNTs This result indicated the effect of titanate nanotubes on the corrosion resistance gradually be increasing percentage of Titanite nanotube as the recorded result. The incorporation of Nanotube material into polyurethane coatings results in the development of a spatial network structure Fig. 7, This structural integrity significantly improves the coating’s ability to resist corrosion by creating barriers that inhibit the penetration of corrosive agents^37^. In this study, the salt spray test results revealed that PU nanocomposite coatings exhibited remarkable corrosion resistance in comparison to the pure PU coatings. This result was attributed to the presence of titanate nanotubes; the nanotubes served as an inert lamellar pigment and consequently prevented corrosion by the formation of a barrier layer against water and oxygen via the parallel alignment to the substrate. This result was in good agreement with that reported previously^15^. Figure 7 shows the schematic of the corrosion inhibition mechanism using titanate nanotubes. Notably, the tubular form of titanates served as an effective laminated barrier, where spherical nanoparticles permitted the passage of aggressive species easily.

**Table 3:** Salt spray resistance of blank polyurethane and Na-TNTs coated panels.

Nanoparticles % in the composite	Exposure time (hours)	Disbanded area %	Rating Number (ASTM D1654)
**Blank polyurethane**	500	19	5
**0.5**	500	9	6
**1.0**	500	7	6
**1.5**	500	5	7
**2.0**	500	5	7
**2.5**	500	2	9

**Fig. 8:**
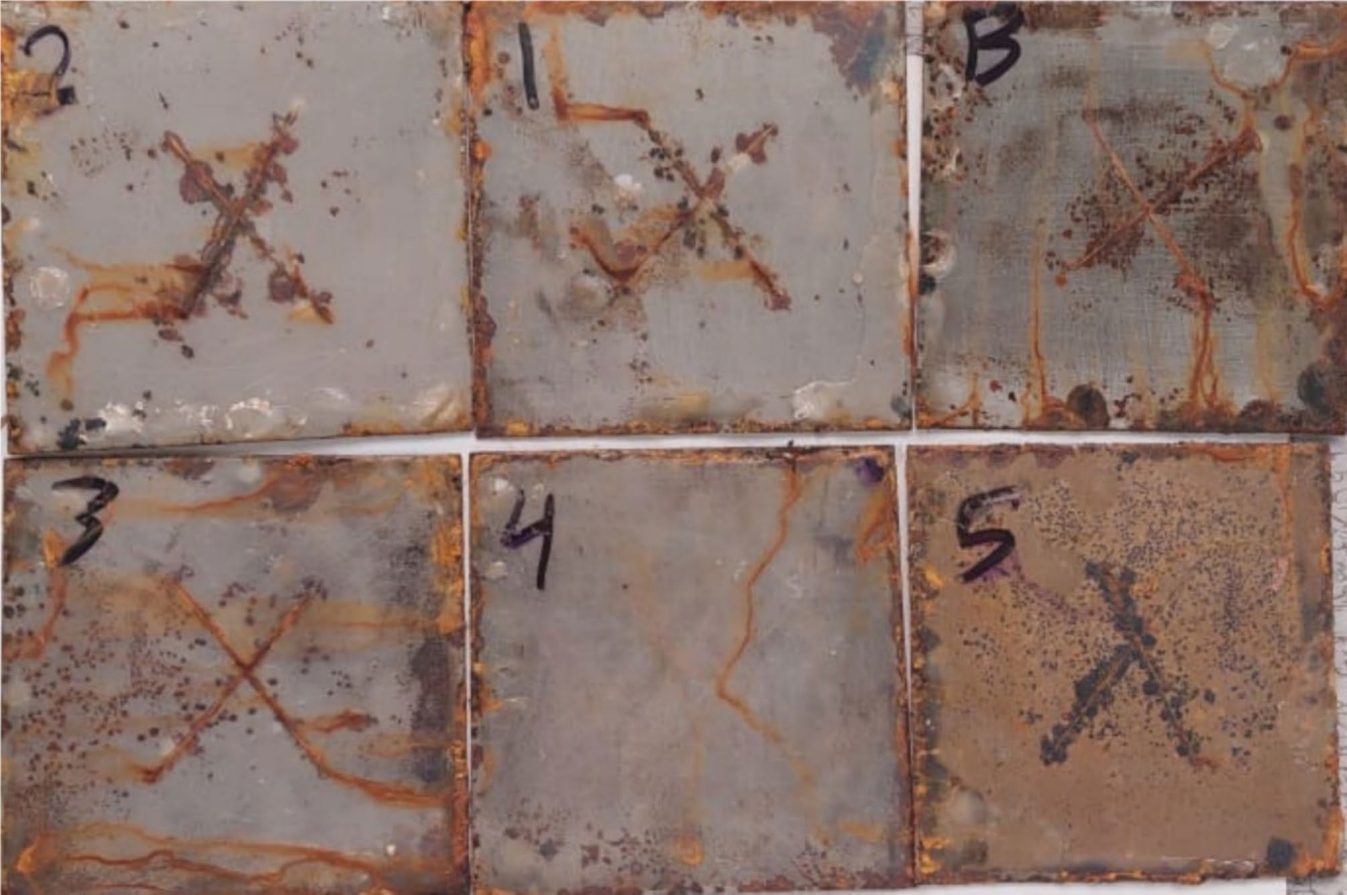
Salt spray tests, where B: blank, 1, 2, 3, 4, and 5 reflect the percentage of the chitosan added to the PU– Na-TNTs.

**Fig. 9:**
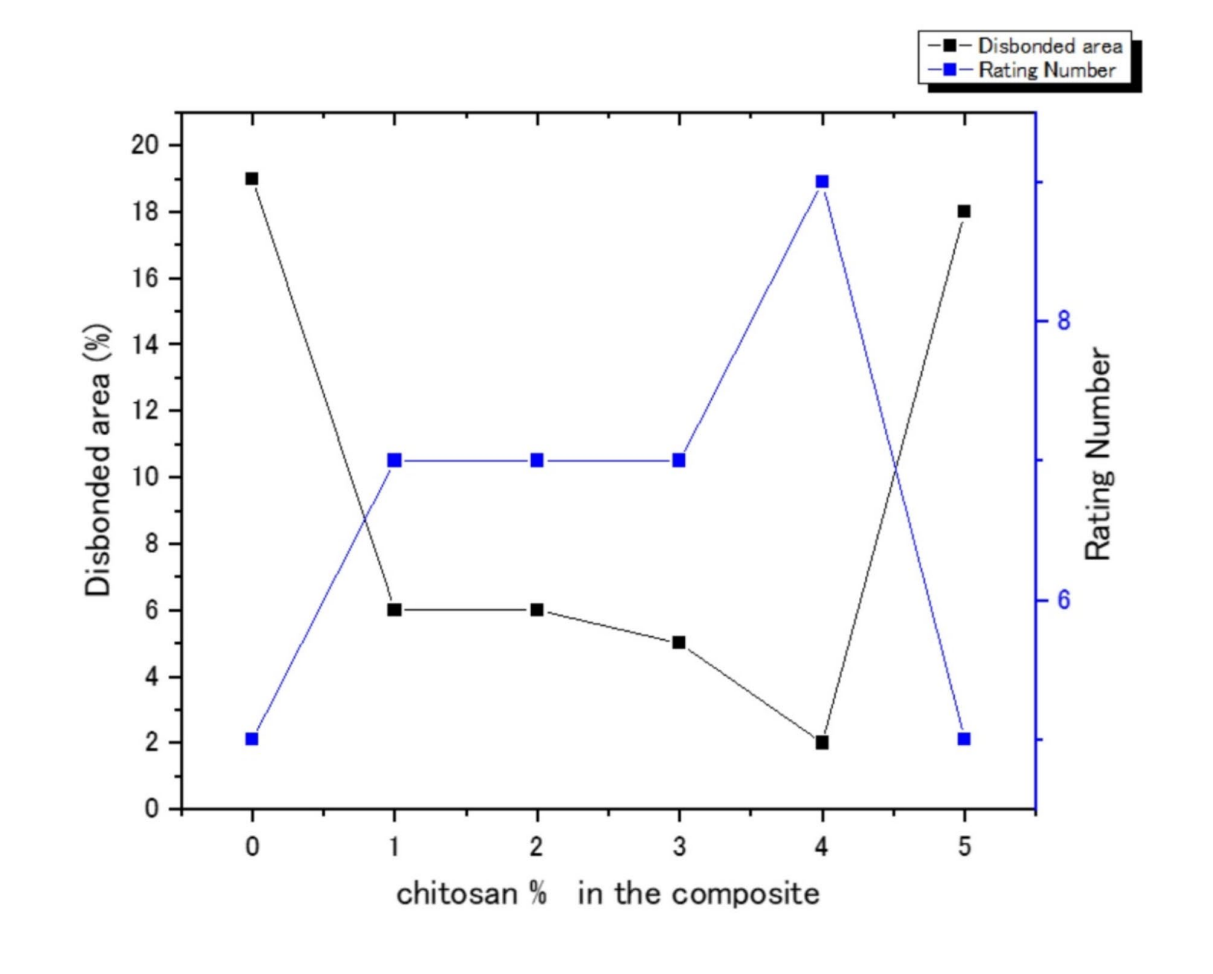
Effect of the chitosan content (%) on the disbonded area.

Figure 8 show the behavior of corrosion by the salt spray test after the addition of chitosan with different ratios to 1.5% Na-TNTs PU, the assessment of figure also implemented according the previous ASTM, and the obtained data are recorded in Table 4; Fig. 9. The percentage of the disbonded area was 6% for 1% and 2% chitosan, and the best results of 2% was realized by using 4% chitosan. However, by using 5% chitosan, the corrosion resistance was suppressed, and the disbonded area revealed 18%, which is revealing to blank polyurethane behavior.

That is, the addition of chitosan to the PU network affected the arrangement and interactions of the polymer chains. As a result, the properties of the new composite were altered in comparison to blank PU. The magnitude and direction of the changes were significantly related to the size, amount, and interaction strength of the chitosan particles^5,7,16–33^. The percentage of 4% chitosan in PU Na-TNTs demonstrated the greatest corrosion resistance and the least corrosion propagation. It showed strong cohesive forces in the film and adhesion between the substrate and the nanocomposite film, which contributed to its superhydrophobic properties and the ability to resist corrosive ions^42^. This result attributed to the good adhesion between the coating and substrate which was in good agreement to the observed pull-off test 7.9 MPa as recorded in Table 2, higher barrier properties, high cross-linked density, and self-healing of chitosan. As showed in Fig. 1 (c, d, e, and f) the optimum result 4% due do good distribution of nanotube and chitosan.

Raising the chitosan content in polyurethane nano composites result in particle agglomeration as shown in Fig. 1 (k, l), which affecting the characteristics of protective coatings. This phenomenon can be attributed to the hydrophilic characteristics of chitosan and increases the viscosity of the mixture, thereby complicating the maintenance of an even distribution of the particles^3,35^. The findings suggest a lack of effective anticorrosive properties. The areas of low density were readily compromised by the salt fog solution, leading to the degradation of cross-linked bonds and the formation of surface pits and corrosion areas on the coating^42^. These changes possibly led to structural changes in the synthesized composites, including increased cavitation, particle aggregation, and restricted matrix chain movement. Furthermore, some defects, including holes and cracks in the film, may be formed around filler particles as indicated in Fig. 1 (k, l), which can impede stress transmission^5,7,16–33^. Hence, for ternary composites (i.e., PU–Na-TNTs–chitosan), the synthesized clusters of titanate and chitosan were immobilized on the material surface^34^, which improved the corrosion resistance of the fabricated coatings and rendered strong adhesion of PU to the substrate surface.

Table 5, summarized the obtained results from corrosion studies for polymer-based nanocomposites for anti-corrosion protection. Our results showed that our composites are more better, since all properties were enhanced, while in the old reported data the composites showed enhancement in certain parameters only.

**Table 4:** Salt spray resistance of polyurethane and chitosan coated panels.

chitosan % in the composite	Exposure time (hours)	Disbanded area %	Rating Number (ASTM D1654)
**Blank**	500	19	5
**1.0**	500	6	7
**2.0**	500	6	7
**3.0**	500	5	7
**4.0**	500	2	9
**5.0**	500	18	5

**Table 5:** Summarize the obtained results for similar composites.

Metal substrate	Polymer	Type of nano material	Shape	Corrosion resistance	Mechanical or other properties	Refrences
Mild steel	Polyurethane	Unmodified and modified ZnO	Spherical	Enhanced corrosion resistance	Hardness, impact resistance, abrasion resistance, storage modulus,	[43]
Carbon steel	Acrylic resin	Unmodified, modified, and graphene grafted CeO2	Flake-like	Enhanced corrosion resistance		[43]
Steel	Polyurethane	Modified and unmodified GO	Nanosheet	Enhanced corrosion resistance	Adhesion improved	[43]
stainless steel	Polyurethane	multiwalled carbon nanotubes (MWNTs)	Nanotube	a good chemical stability in a corrosive environment	enhanced thermal stability	[1]
Phosphated steel	Polyurethane	multiwalled carbon nanotubes (MWNTs)	Nanotube	Enhanced corrosion resistance	Enhanced adhesion	[44]
Aluminum alloy	Polyurethane	multiwalled carbon nanotubes (MWNTs)	Nanotube	Enhanced corrosion resistance	Enhanced adhesion	[44]
Steel	Epoxy	Titanium oxide nanotubes	Nanotube	Enhanced corrosion resistance	Enhanced Mechanical and thermal properties	[15]

### Thermal stability

**Fig. 10:**
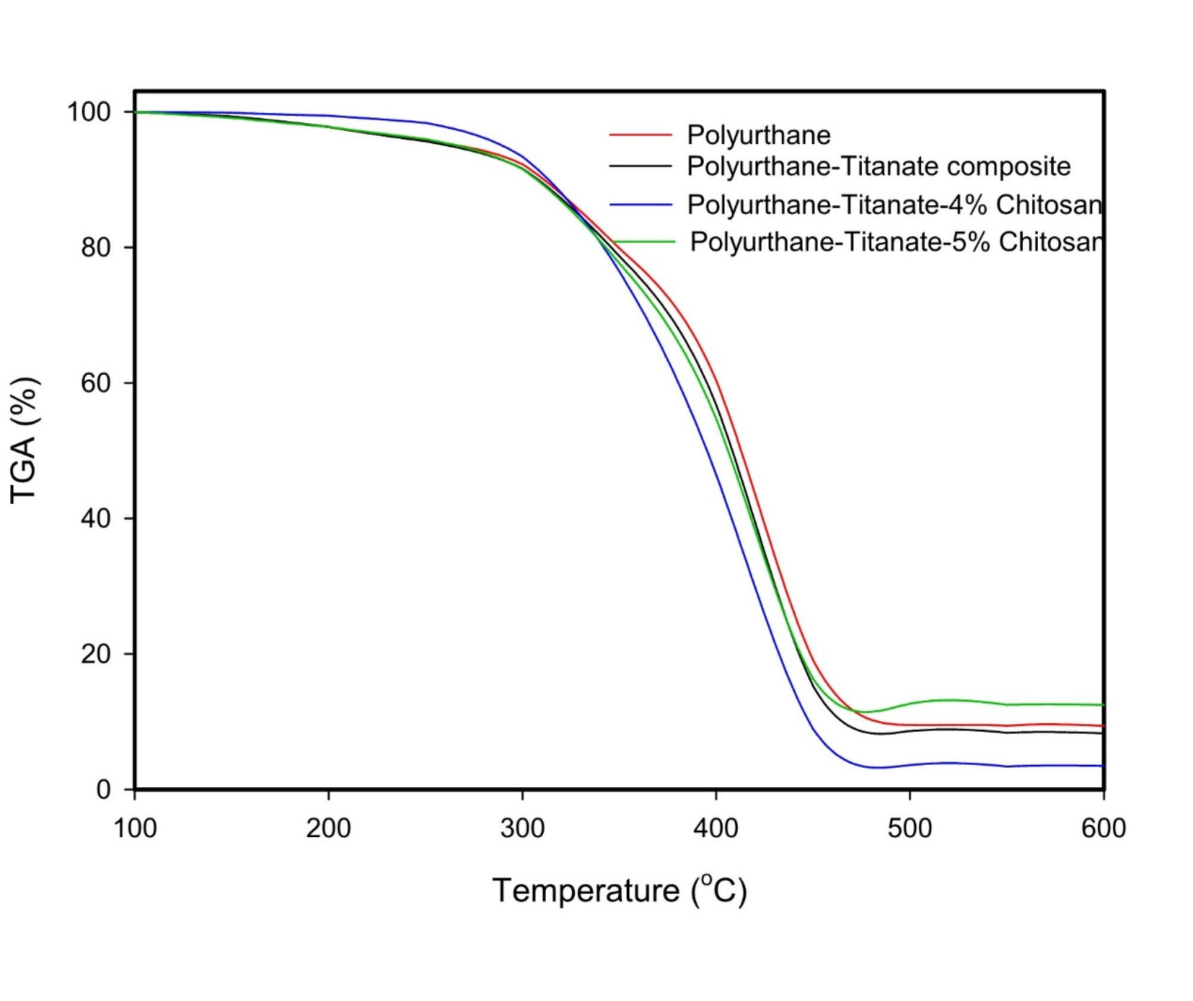
Thermal gravimetrical analysis (TGA) of polyurethane, polyurethane – Na-TNTs, composite containing 4 and 5% chitosan.

The thermal gravimetric analysis data in Fig. 10 shows that the incorporation of 4% chitosan in PU improve the thermal stability at 250 compared to blank PU, PU Na-TNTs and 5% chitosan PU Na-TNTs.

Augmenting the proportion of chitosan in PU formulations is associated with improved thermal characteristics, signifying intensified intermolecular interactions via hydrogen bonding between PU and chitosan^35^.

Potential application:

In settings where elevated temperatures align with corrosive conditions, these nanocomposites can function as protective coatings for metal substrates, enhancing durability and longevity, Anti-Microbial coating, Environmental Applications,

## Conclusions

In this study, a titanate–polyurethane–chitosan ternary nanocomposite was synthesized by physical mixing, and sodium-titanate nanotubes (Na-TNTs) were synthesized by the hydrothermal method. The nanosized Na-TNTs were obtained with an average diameter of 10.7 nm, and microstructural images revealed that the added chitosan was homogeneously distributed in the PU matrix. Mechanical tests were conducted according to the ASTM procedures for organic coating on steel. The results of the adhesion strength, impact resistance, hardness, and abrasion tests of the cured blank PU and modified PU (addition of nanoparticles and chitosan) revealed that the addition of Na-TNTs and chitosan significantly improved the mechanical properties. By the addition of chitosan, the chain order of the PU network increased, and the mechanical properties of the composite changed in comparison to pure PU.

## Data Availability

All data generated or analysed during this study are included in this published article.
